# Cat ownership is associated with increased asthma prevalence and dog ownership with decreased spirometry values

**DOI:** 10.1590/1414-431X20187558

**Published:** 2018-10-18

**Authors:** C.S. Simoneti, E. Ferraz, M.B. Menezes, T.R. Icuma, E.O. Vianna

**Affiliations:** 1 Departamento de Clínica Médica, Faculdade de Medicina de Ribeirão Preto, Universidade de São Paulo, Ribeirão Preto, SP, Brasil; 2 Departamento de Fisioterapia, Centro Regional Universitário de Espírito Santo do Pinhal, Espírito Santo do Pinhal, SP, Brasil; 3 Departamento de Medicina Social, Faculdade de Medicina de Ribeirão Preto, Universidade de São Paulo, Ribeirão Preto, SP, Brasil

**Keywords:** Respiratory tract diseases, Animals, Bronchial hyperreactivity, Lung function, Pets

## Abstract

The association between pet ownership and the development of allergic and respiratory diseases has been the aim of several studies, however, the effects of exposure in adults remain uncertain. The aims of the present study were to investigate the prevalence of asthma and lung function status among dog and cat owners. This cross-sectional study was performed at two universities with students and workers who were allocated into 3 groups according to pet ownership in the previous year: cat owners, dog owners, and no pets (control group). Subjects underwent spirometry, bronchial challenge test with mannitol, skin prick tests, and questionnaires about animal exposures and respiratory symptoms. Control group comprised 125 subjects; cat owner group, 51 subjects; and dog owner group, 140 subjects. Cat owners had increased asthma prevalence (defined by symptoms and positive bronchial challenge test), but no changes in lung function compared to the control group. The dog owner group had lower spirometry values (forced expiratory volume in one second and lower forced vital capacity), but similar asthma prevalence, compared to the control group. In the cat owner group, excess of asthma may have an immunological basis, since we found an association with atopy. Although we did not have endotoxin data from volunteers' households, we postulated that low values of lung function were associated to exposure to endotoxins present in environments exposed to dogs.

## Introduction

The effect of exposure to pets on the respiratory system has long been studied and is still controversial. In the 1990's, it was shown that sensitization to cats was a risk factor for asthma (1). In turn, exposure to cat allergen was associated with increased bronchial responsiveness (2). Subsequently, it was demonstrated that in atopic people, total IgE above 100 kU/L and specific IgE against cats above 0.35 kU/L were associated with new-onset asthma (3). In another study, daytime attacks of breathlessness were associated with cat ownership (4).

However, the same authors found a negative association with having a dog and infections of the airways, thus suggesting a possible protective effect (4). A systematic review involving studies with children and adults concludes that there is not enough evidence to indicate a relationship between keeping a pet and subsequent development of asthma (5). This lack of consensus and the discrepancy of the effects of pets reinforce the need for further studies. Thus, the aim of the present study was to investigate the prevalence of asthma and lung function status among dog and cat owners.

## Material and Methods

The present cross-sectional study was conducted among adults working or studying at two Brazilian universities, the University of São Paulo at Ribeirão Preto campus and the State University of Campinas. For the present analysis, we selected individuals who had owned a cat or a dog as a pet in the year prior to the study; individuals who had not owned pets were selected as the control group. Consequently, the present study comprised three groups: cat owner, dog owner, and control groups. Subjects had no work exposure to laboratory animals or other animals.

Subjects answered a questionnaire and underwent a skin prick test (SPT), pulmonary function test, and bronchial challenge test with mannitol. Lung function was measured using a Koko spirometer and software (PDS Instrumentation, Inc., USA). Measurements were performed in the sitting position with the volunteer wearing a nose clip. At least 3 technically satisfactory maneuvers were attempted for each volunteer. If at least 3 technically satisfactory maneuvers were not obtained after 8 attempts, lung function testing was discontinued.

In order to perform the bronchial challenge test, an inhalation device and capsules (Pharmaxis Ltd., Australia) containing dry powdered mannitol were used. The challenge began with an empty capsule (used for training), followed by inhalation of 5, 10, 20, 40, 80, 160, 160, and 160 mg of dry powdered mannitol. Forced expiratory volume in one second (FEV_1_) was measured 60 s after every inhalation. This procedure was repeated for each dose until inhalation of 635 mg of mannitol or 15% fall in FEV_1_. The bronchial challenge test was considered positive when a 15% decrease in FEV_1_ was observed, indicating bronchial hyperresponsiveness (BHR). An individual was considered to be an asthmatic subject if he or she had BHR and had experienced symptoms of wheezing, chest tightness during the night, or dyspnea during the day or at night in the previous 12 months.

Subjects underwent SPT with 11 allergens. A wheal diameter of at least 3 mm was considered positive. The volunteers also responded to a questionnaire adapted from the European Community Respiratory Health Survey (ECRHS), translated and tested in Brazil. Further information on all the procedures performed can be found elsewhere (6).

### Statistical analysis

Univariate analysis (chi-squared test) was performed to compare prevalence of asthma among control, and cat and dog owner groups. ANOVA was used to compare age and spirometric data (continuous variables) among groups. Relative risk (RR) was estimated using a modified Poisson regression approach, i.e., Poisson regression with a robust error variance. The model was adjusted using the Package Geepack in the R Software (R Foundation for Statistical Computing, Austria), version 3.2.2 and confidence intervals for the RR were obtained with the Package doBy in the R Software (R Foundation for Statistical Computing). Linear regression was used to make predictions about forced vital capacity (FVC) and FEV_1_.

## Results

A total of 316 volunteers were included. Of these, 125 had not been pet owners in the previous year (control group), 51 individuals reported having had a cat, and 140 subjects reported having had a dog. Table 1 presents the general characteristics and the outcomes studied. The group of individuals exposed to dogs had lower FEV_1_ (reported in percentage of predicted values) and lower forced vital capacity (in percentage of predicted values) than the control group.

**Table 1:** Characteristics and outcomes of the study sample.

	Control group (n=125)	Cat owner group (n=51)	Dog owner group (n=140)	P value
Age	32.5±9.8	32.8±11.4	32.0±10.3	0.844
Sex				
Female	83 (66.4%)	34 (66.7%)	97 (69.3%)	0.868
Male	42 (33.6%)	17 (33.3%)	43 (30.7%)	0.868
Smoking	13 (10.4%)	10 (19.6%)	25 (16.7%)	0.151
SPT positive	50 (40.0%)	23 (45.1%)	71 (50.7%)	0.216
Confirmed asthma	6 (4.8%)	8 (15.7%)	15 (10.7%)	0.053
FEV_1_ (%)	99.4±13.1	99.6±13.4	96.0±10.1	0.035*
FVC (%)	100.4±13.2	101.5±13.5	97.2±10.8	0.038*

Data for age, FEV1, and FVC are reported as means±SD. SPT: skin prick test; FEV_1_: forced expiratory volume in 1 s (% of predicted value); FVC: forced vital capacity (% of predicted value). Statistical analysis was done with chi-squared test. *Pairwise comparisons are shown in Table 3.

Multivariate analysis showed that individuals exposed to cats had increased risk of confirmed asthma compared to individuals in the control group (RR: 3.24; 95% confidence interval (CI): 1.31 to 7.99) (Table 2). Linear regression showed that dog exposure was associated with lower FVC in percentage of predicted values (adjusted coefficient: –3.61, 95%CI: –6.62 to –0.60) and lower FEV_1_ in percentage of predicted (adjusted coefficient: –3.50, 95%CI: –6.45 to –0.55) compared to the control group. Cat ownership was not associated with changes in lung function (Table 3).

**Table 2:** Assessment of risk factors for confirmed asthma.

Risk factors	Crude model				Adjusted model			
	RR	95%CI		P value	RR	95%CI		P value
Group (cat *vs* control)	3.28	1.20	8.97	0.02	3.24	1.31	7.99	0.01
Group (dog *vs* control)	2.21	0.89	5.52	0.08	1.74	0.73	4.16	0.21
Age (continuous)	0.94	0.89	0.99	0.01	0.96	0.91	1.01	0.09
Sex (female *vs* male)	0.66	0.29	1.48	0.31	0.54	0.25	1.15	0.10
SPT (positive *vs* negative)	15.75	3.81	65.09	<0.001	14.12	3.58	55.69	<0.001
Smoking (yes *vs* no)	0.42	0.10	1.70	0.22	0.58	0.15	2.26	0.43

RR: relative risk; CI: confidence interval; SPT: skin prick test.

**Table 3:** Assessment of risk factors for reduction in FVC and FEV_1_.

Risk factors	FVC				FEV_1_			
	Adjusted coefficient	95%CI		P value	Adjusted coefficient	95%CI		P value
Group (cat *vs* control)	0.28	–3.77	4.34	0.89	–0.15	–4.13	3.82	0.94
Group (dog *vs* control)	–3.61	–6.62	–0.60	0.01	–3.50	–6.45	–0.55	0.02
Age (continuous)	0.09	–0.05	0.24	0.20	0.02	–0.12	0.17	0.73
Sex (female *vs* male)	–1.65	–4.67	1.37	0.28	–1.45	–4.41	1.51	0.33
SPT (positive *vs* negative)	0.44	–2.39	3.26	0.76	–1.85	–4.62	0.92	0.19
Smoking (yes *vs* no)	4.28	0.14	8.42	0.04	2.27	–1.78	6.33	0.27

FVC: forced vital capacity (% of predicted value); FEV_1_: forced expiratory volume in 1 s (% of predicted value); CI: confidence interval; SPT: skin prick test.

## Discussion

The risks and/or benefits of keeping a pet have been the subject of several studies. This knowledge becomes even more important for regions or countries with relevant prevalence of pet ownership, such as the United Kingdom (7) and Italy (8). Pets (dogs or cats) are found in 9% of households of asthmatics in Spain (9).

Data from the present study showed that cat ownership was associated with increased risk of asthma but not with reduced lung function, while dog ownership was associated with reduced pulmonary function (FEV_1_ and FVC), without an increased risk of asthma. Previous studies have shown that exposure to cats is associated with increased risk of asthma among adults (10,11), however, to our knowledge, this is the first study to show concomitantly that dog ownership is associated with changes in lung function in adults. It is noteworthy that although we found a statistically significant reduction of lung function in dog owners, such reduction was not clinically worrisome. Reductions in spirometric values should be taken with caution. Values were still in the normal range and changes were small, approximately, 3.5% of predicted value. This fall is not a reason for concern among the general population; however, the change is important due to its mechanisms, its implications in patients with severe respiratory disease, and its relevance for disease understanding.

We believe that the increased risk of asthma among individuals exposed to cats is associated with immunological mechanisms because, as expected, atopy was a risk factor for asthma. In turn, changes in lung function of individuals exposed to dogs were not associated with atopy. It has been shown that households in which there are dogs have greater levels of airborne endotoxins (12) and exposure to endotoxins may worsen lung function (13,14).

Among sewage treatment plant workers, low levels of endotoxin had a significant impact on the observed across-shift (during 6 h shift) decline in FEV_1_ (13). In another study, workers of a factory that produced bioproteins derived from a bacterial species (*Methylococcus capsulatum*), exposed to low concentrations of endotoxin, presented lower FVC during the period of occupational exposure, compared to the period without occupational exposure. In addition, the authors detected increased leukocyte count during the exposure period (14). These findings reveal the association between exposure to endotoxin and inflammatory activity in the workers' lungs.

In urban areas, pets frequently live in apartments with no sufficient ventilation and upholstered furniture and carpets, where the level of exposure to pet allergens is very high (15). In residences not exposed to dogs and cats in the previous 6 months, the geometric mean concentrations of dog and cat allergens were 1.33 μg/g and 1.47 μg/g, respectively. In households exposed to dogs and cats, the geometric mean concentrations were 69.23 μg/g and 199.70 μg/g, respectively (16). Threshold values generally recognized as sufficient to induce sensitization and trigger respiratory symptoms are, respectively, 1 μg and 8–10 μg of allergen/g of dust (17). Thus, concentrations of dog and cat allergens in households not exposed to such animals would be insufficient to induce symptoms. In addition, exposure to animals is associated with the presence of other agents, such as the endotoxins, previously mentioned (12). In a recent study to evaluate home fungal and bacterial microbiomes, bacterial evenness and diversity, but not richness, were significantly increased by the presence of a dog, but not by the presence of a cat. Homes with dogs contained four times more putative bacterial species compared to homes without a dog (18).

Our data demonstrated that exposure to cats was associated with increased risk of asthma, while exposure to dogs is associated with reduced lung function. We speculate that the outcomes have different pathways for their development. Asthma would have an immunological basis (marked by atopy as a risk factor), and there may be a latency period between the onset of cat exposure and the development of asthma. On the other hand, reduction of pulmonary function can be associated with endotoxin exposure, which causes inflammation and may rapidly lead to changes in lung function.
